# Author Correction: Enhanced super-resolution microscopy by extreme value based emitter recovery

**DOI:** 10.1038/s41598-021-02193-3

**Published:** 2021-11-16

**Authors:** Hongqiang Ma, Wei Jiang, Jianquan Xu, Yang Liu

**Affiliations:** 1 Biomedical and Optical Imaging Laboratory, Deparstments of Medicine and Bioengineering, University of Pittsburgh, Pittsburgh, PA 15213 USA; 2 Department of Pathology, West China Second University Hospital, Sichuan University, Chengdu, 610041 People’s Republic of China

Correction to: *Scientific Reports* 10.1038/s41598-021-00066-3, published online 14 October 2021

The original version of this Article contained an error in Reference 7, which was incorrectly given as:

Hoogendoorn, E. *et al.* The fidelity of stochastic single-molecule super-resolution reconstructions critically depends upon robust background estimation. *Sci. Rep.* **4**, 3854 (2015).

The correct reference is listed below:

Hoogendoorn, E. *et al.* The fidelity of stochastic single-molecule super-resolution reconstructions critically depends upon robust background estimation. *Sci. Rep.* **4**, 3854 (2014).

As a result, Reference 11 was a duplication of Reference 7 and therefore removed from the Article. The References have been renumbered.

The original Article has been corrected.

