# Supplementary material for: Enhanced super-resolution microscopy by extreme value based emitter recovery
Source: Sci Rep. 2021 Oct 14;11:20417. doi: 10.1038/s41598-021-00066-3 (PMC8517018; doi:10.1038/s41598-021-00066-3)
Supplement: Supplementary file 1 — Supplementary Information. [file 41598_2021_66_MOESM1_ESM.docx]

**Enhanced super-resolution microscopy by extreme value based emitter recovery**

[*Hongqiang Ma*](http://www.ncbi.nlm.nih.gov/pubmed/?term=Ma%20H%5BAuthor%5D&cauthor=true&cauthor_uid=26390959)*^1,*^, Wei Jiang^1,2^, Jianquan Xu^1^ and Yang Liu^1,*^*

*1. Biomedical and Optical Imaging Laboratory, Departments of Medicine and Bioengineering, University of Pittsburgh, Pittsburgh PA 15213, USA*

*2. Department of Pathology, West China Second University Hospital, Sichuan University,* *Chengdu 610041, P.R.China*

**Email:* [*hongqiang.ma@pitt.edu*](mailto:hongqiang.ma@pitt.edu) *&* [*liuy@pitt.edu*](mailto:liuy@pitt.edu)

**Supplementary Information**

**Supplementary Methods**

We also validate the accuracy of EVER against the ground truth using simulated dataset for each of the image characteristics contributed by heterogeneous background structures, emitter density, size and intensity. Table S1 lists the set of parameters used in each simulation, including four background structures with a sinusoidal shape (spatial frequency of the sine cycle = 0, 2, 6, 10), four emitter density levels (1, 3, 5, 10 emitters/µm^2^), three emitter sizes (σ_PSF_ =1, 2, 3 pixels) and four emitter intensity levels (1000, 5000, 10000, 15000 photons). The total photon number of the molecules is set to be 5000 to mimic the commonly used fluorophore Alexa Fluor 647 and the background photon number is set to vary from 200 to 800 to mimic the conditions in most biological experiments.

**Table S1.** List of parameters of different image characteristics.

| **Simulation parameters** | **Figure 2** | **Figure 3** | **Figure S1** | **Figure S2** | **Figure S3** | **Figure S4** | **Figure S5** | **Figure S6** |
| --- | --- | --- | --- | --- | --- | --- | --- | --- |
| **Image size**  **(pixels)** | 128x128 | 64x32 | 128x128 | 128x128 | 128x128 | 128x128 | 64x64 | 64x64 |
| **Frame number** | 100 | 1000 | 100 | 100 | 100 | 100 | 100 | 100 |
| **Pixel size**  **(nm)** | 100 | 100 | 100 | 100 | 100 | 100 | 100 | 100 |
| **Emitter density (emitters/µm^2^)** | 5 | 1 | 3 | 1, 3, 5, 10 | 1 | 100 | 3 | 2 |
| **Emitter size**  **(σ_PSF_, pixel)** | Lognormal  mean:1.5  std: 0.4 | Left: 1, middle: 2 right:3 | 1 | 1 | 1, 2, 3 | 1 | 1~3 | 1 |
| **Emitter intensity**  **(photons)** | lognormal  mean:5000  std: 2000 | Left: 5000, middle: 10000 right:15000 | 5000 | 5000 | 5000 | 2 | 5000 | 100~300 |
| **Background structure (intensity, type)** | 500±300, spatial frequency 10 sine cycles per image | 500±300, spatial frequency (sine cycles per image) = 2 | 500±300, spatial frequency (sine cycles per image) = 0, 2, 6, 10 | 500, uniform | 500, uniform | 1000, 5000, 10000, 15000 | 500±300, spatial frequency (sine cycles per image) = 4 | 30±10, spatial frequency (sine cycles per image) = 4 |

**Supplementary Figures**

*A. Heterogeneous background*

Heterogeneous background is often present when imaging biological samples, especially in thick cells and tissue section. We simulate a series of imaging dataset in the presence of both uniform and heterogeneous background whose spatial variation is modeled by different sinusoidal shapes. Figure S1(a) shows the raw image (left column), true background and the estimated background based on EVER, MED and RB. Figure S1(b) shows the corresponding cross-sectional profile of the estimated background and the true background (TRUE). The background estimated by EVER (red lines) shows the closest match with the true background; the background estimated by MED (green lines) shows apparent over-estimation, as indicated by the higher intensity in the regions with emitters in Fig. S1(b); the spatial filter of RB (blue lines) shows significant over- and under-estimation depending on the structures of the heterogeneous background, especially in the regions with a higher spatial frequency.


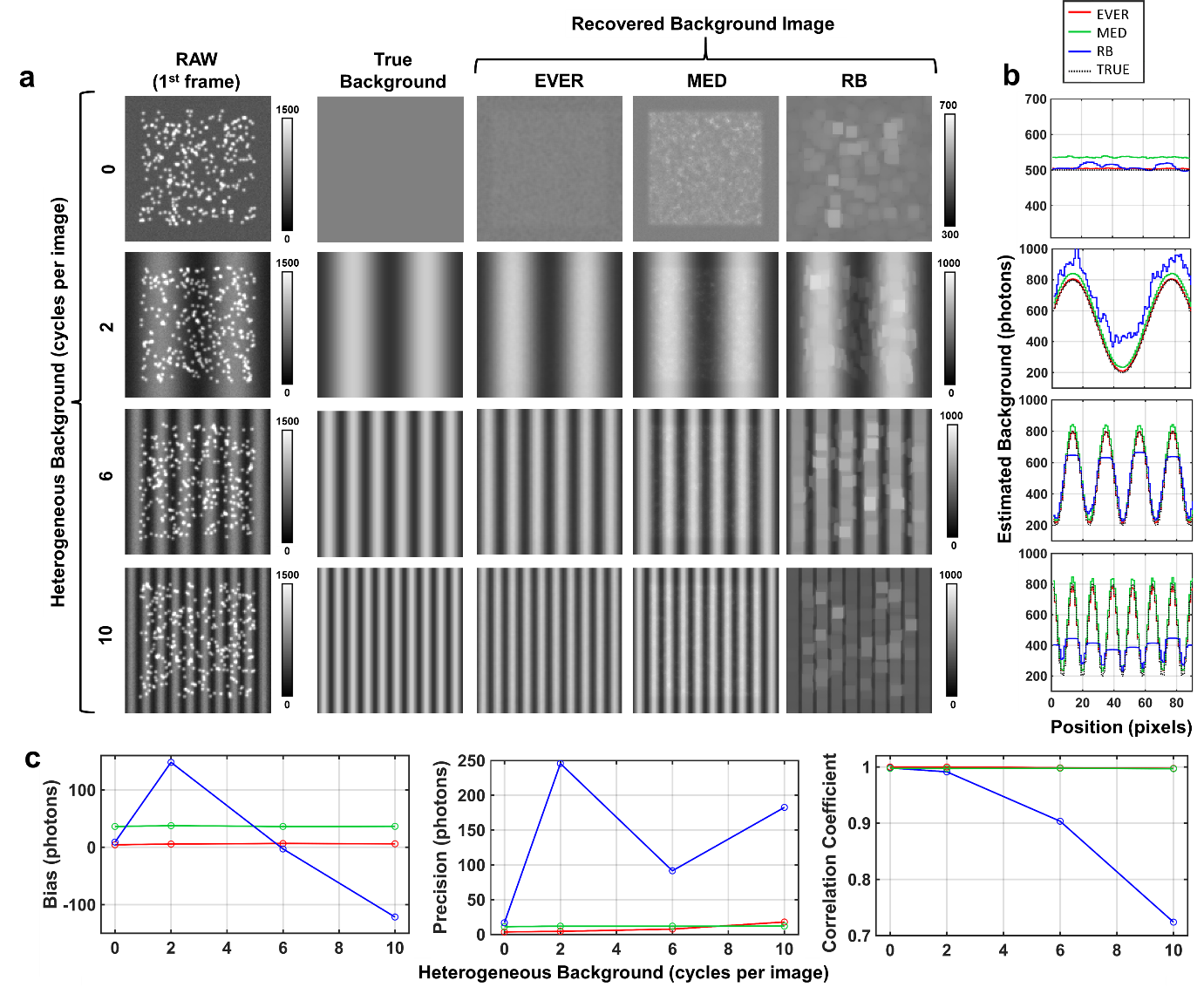


**Figure S1.** (a) Estimated background for various heterogeneous background using 3 different algorithms (EVER, MED and RB). The dataset is simulated with a sinusoid-shaped background at a spatial frequency of 0, 2, 6 and 10 cycles per image, with intensity ranging from 200 to 800 photons. (b) Cross-sectional profiles of the background estimated by EVER, MED and RB. Central 90x90 pixels of the image was used for analysis. (c) The background estimation bias, precision and correlation coefficient of EVER, MED and RB for various heterogeneous background.

B. Emitter density

In super-resolution localization microscopy, the emitter density can vary across different imaging regions depends on the density of the imaging target. On the other hand, a mainstream is to use relative high emitter density to improve the temporal resolution and throughput. In this section, we evaluate the effect of emitter density (1, 3, 5 and 10 emitters/µm2) on the accuracy of background estimation. As shown in Figs. S2(a-b), the background estimated by EVER shows little over-estimation and closest match with the ground truth even for the high-density scenario (5 and 10 emitters/µm2); MED shows significant over-estimation and becomes significantly worse in the high-density scenarios; and RB shows significant over-estimation and also introduces heterogeneous background. The quantitative metrics shown in Fig. S2(c) also confirms our observed performance characteristics. The EVER-corrected image shows the highest image correlation coefficient (>99.9%) with the ground truth. When the emitter density is <3 emitters/µm^2^, the estimation bias and precision of EVER are <5 photons compared to the ground truth. Even at a high emitter density of 10 emitters/µm^2^, EVER slightly over-estimates the background only by < 30 photons, significantly lower (~5 and 10 times) than that by RB and MED. In contrast, even at a moderate emitter density of 3 emitter/µm^2^, MED over-estimates the background by >40 photons. It becomes even worse at a high emitter density of 10 emitter/µm^2^, over-estimating the background by ~400 photons. On the other hand, RB–as a spatial filter–cannot well distinguish foreground from background, and the estimated background shows the worst precision. These results demonstrate that EVER-based background correction maintains the high level of image fidelity and robustness in both sparse and high-density single-molecule localization scenarios, but the performance of other conventional methods becomes dramatically worse in the high-density scenarios.


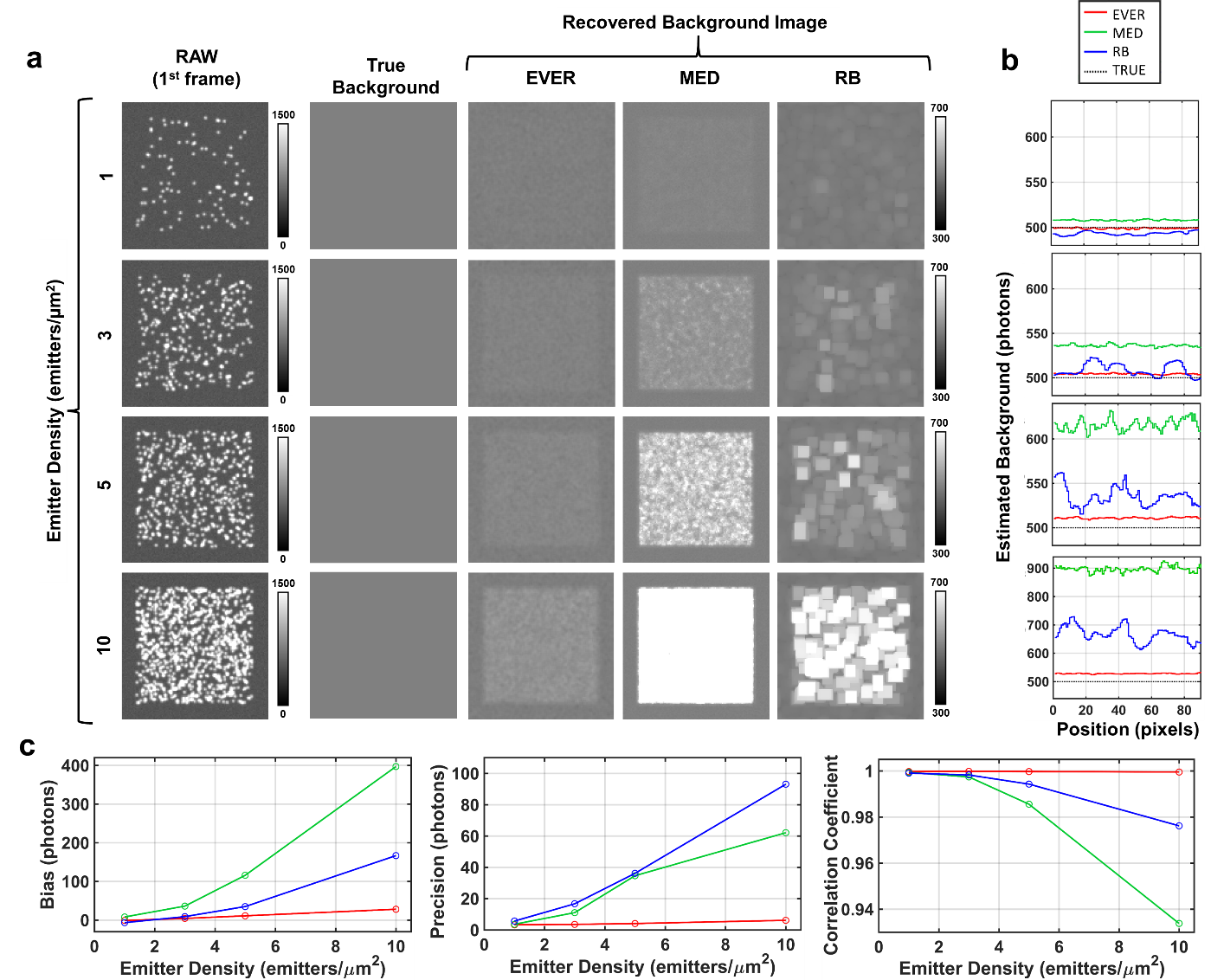


**Figure S2.** (a) The estimated background using EVER, MED and RB for various emitter densities (1, 2, 5 and 10 emitters/µm^2^). The simulated dataset assumes a uniform background of 500 photons per pixel. (b) Cross-sectional profiles of the background image estimated by EVER, MED and RB for various emitter densities. Central 90x90 pixels of the image were used for analysis. (c) The background estimation bias, precision and correlation coefficient of EVER, MED and RB for various emitter densities.

*C. Emitter size*

Emitter size is especially important for 3D localization microscopy where the size is often used to derive the axial location of each emitter. In this section, we compare the performance of EVER, MED and RB to estimate background for dataset with different emitter size quantified by the standard deviation (sigma) of emitter’ point spread function (1, 2 and 3 pixels), as shown in Supplementary Fig. S2. Overall, EVER shows the closest match against the ground truth and the best accuracy to estimate background for all simulated emitter sizes, with the estimation bias of <10 photons, the estimation precision of EVER <5 photons and the correction coefficient >99.1% against the ground truth (shown in Supplementary Fig. S2). In contrast, both MED and RB suffer from different degrees of over-estimation with significantly worse precision and correlation coefficient against the ground truth. Therefore, these results demonstrate that EVER-based background correction can accurately recover emitters with different sizes, even for those larger emitters that are slightly off the focus that is often difficult to recover by other methods.

**
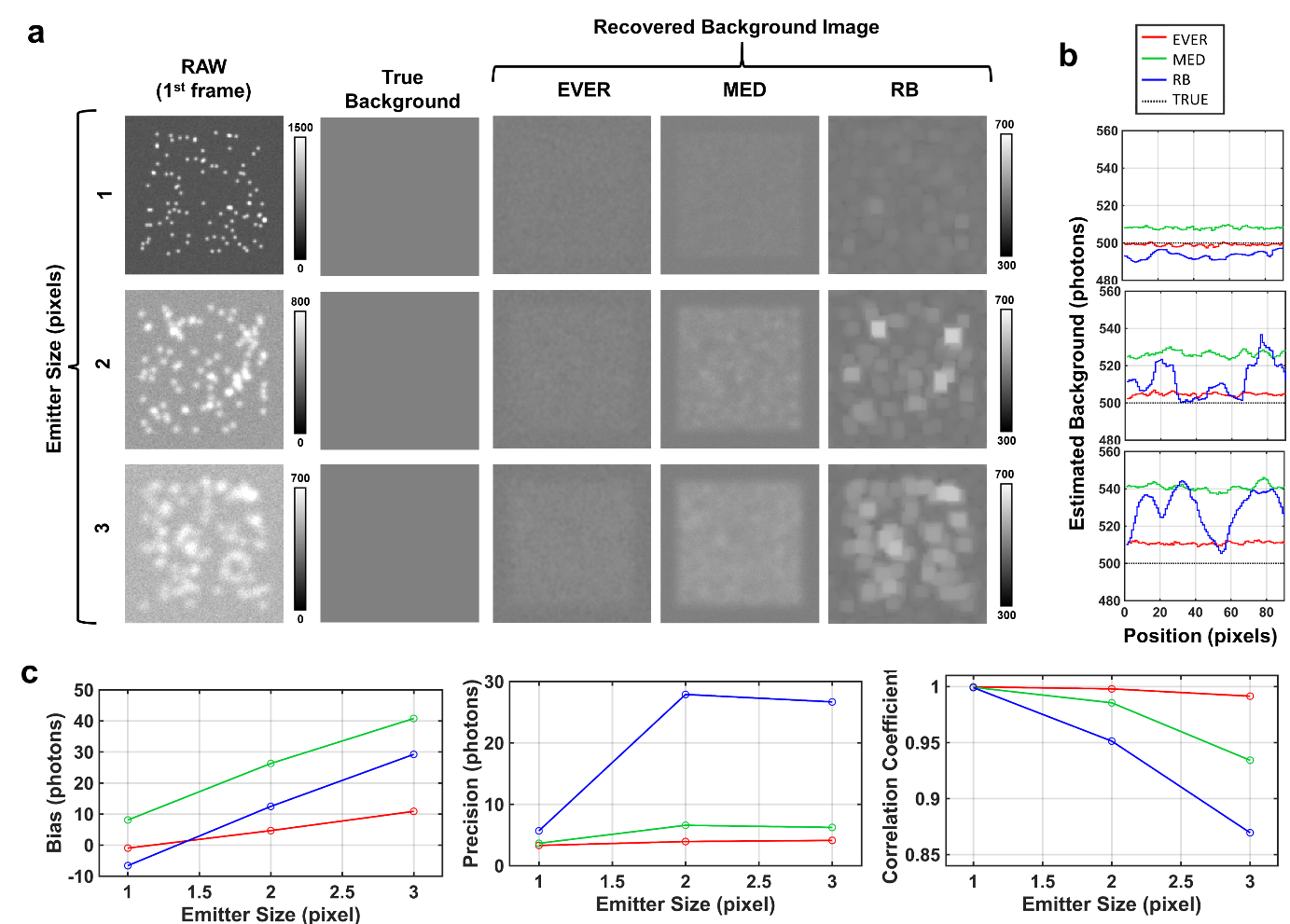
**

**Figure S3.** (a) The estimated background by EVER, MED and RB for various emitter sizes (standard deviation of emitter’s PSF = 1, 2 and 3 pixels). The simulated dataset assumes a uniform background of 500 photons per pixel and the total photon per molecule is 5000. (b) Cross-sectional profiles of the background image estimated by different algorithms for various emitter size. Central 90x90 pixels of the image was used for analysis. (c) The background estimation bias, precision and correlation coefficient of EVER, MED and RB for various emitter sizes with the ground truth.

*D. Emitter intensity*

Emitter intensity is an important characteristic that impacts localization accuracy. In this section, we compare the performance of background correction by EVER, MED and RB for dataset with different emitter intensity (or photon number) that ranges from 1000 to 15000 photons. Similarly, EVER shows the best performance for all cases with the estimation bias of <8 photons, estimation precision of <5 photons and image correlation coefficient of > 99.1% against the ground truth, significantly better than those estimated by MED and RB (shown in Supplementary Fig. S3). These results demonstrate that EVER-based background correction shows the best accuracy for both weak and bright emitters.

**
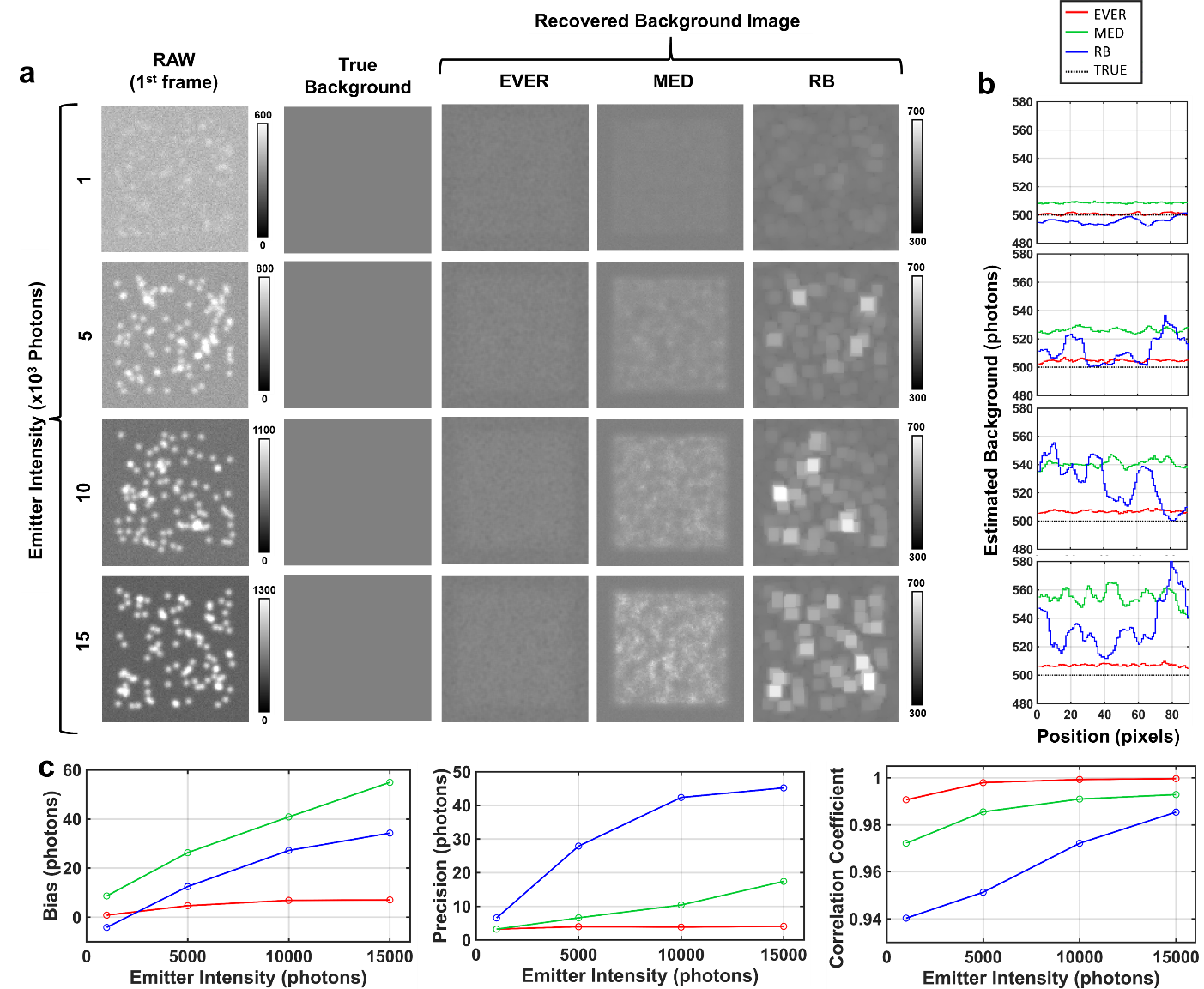
**

**Figure S4.** (a) The background estimation by EVER, MED and RB for various emitter intensities. The simulated dataset assumes a uniform background of 500 photons per pixel and the total photon per emitter ranges from 1000 to 15000. (b) The profiles of the estimated background image by EVER, MED and RB for dataset with various emitter intensities. Central 90x90 pixels of the image was used for analysis. (c) The background estimation bias, precision and correlation coefficient of EVER, MED and RB for various emitter intensities.


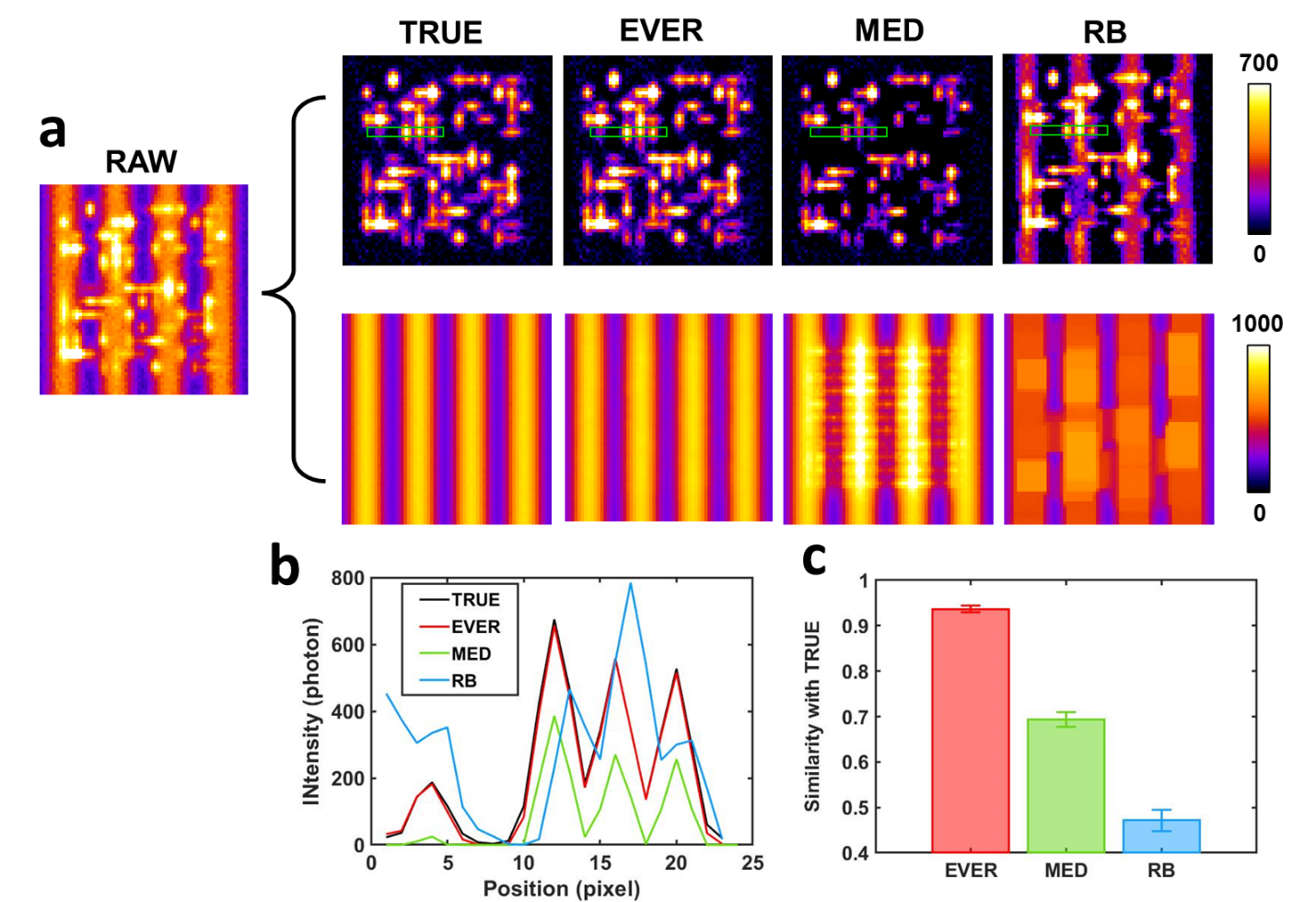


**Figure S5.** A simulated raw image where the emitters with astigmatism point spread function are mixed with heterogeneous background, and the emitter (upper panel) and background images (lower panel) are from ground-truth (TRUE), and those recovered by extreme value-based emitter recovery (EVER), temporal median filter (MED) and spatial rolling ball filter (RB). **(b)** The intensity profile of the region in the green rectangular box of (a). **(c)** Image similarity between the TRUE image and the recovered emitter images using EVER, MED and RB.


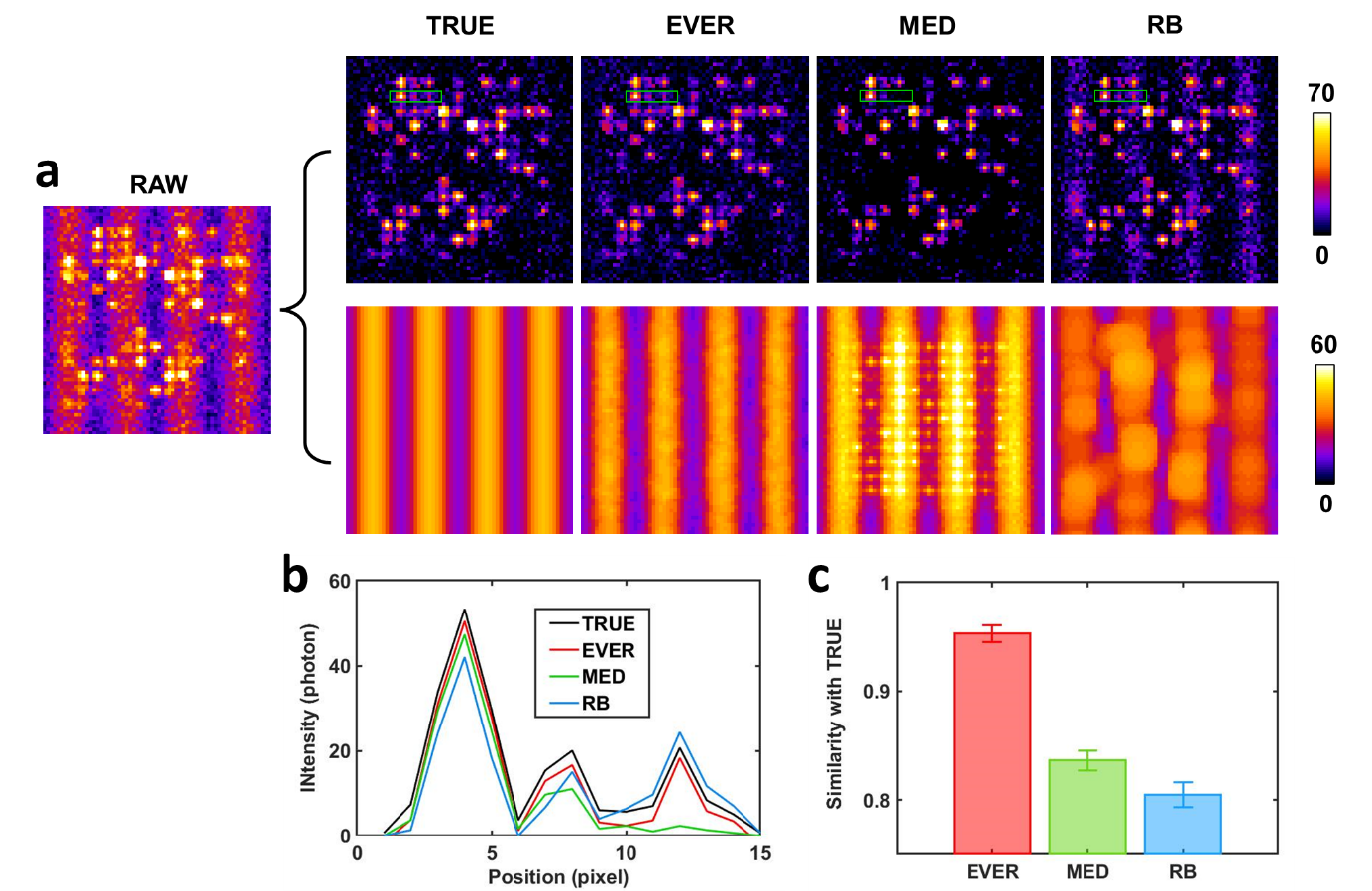


**Figure S6.** A simulated raw image where the dim emitters (100~300 photons) are mixed with heterogeneous background (20~40 photons), and the emitter (upper panel) and background images (lower panel) are from the ground-truth (TRUE), and those recovered by extreme value-based emitter recovery (EVER), temporal median filter (MED) and spatial rolling ball filter (RB). **(b)** The intensity profile of the region in the green rectangular box of (a). **(c)** Image similarity between the TRUE image and the recovered emitter images using EVER, MED and RB.
